# Developmental differences in relations between parent-reported executive function and unitized and non-unitized memory representations during childhood

**DOI:** 10.3389/fpsyg.2015.01214

**Published:** 2015-08-19

**Authors:** Sarah L. Blankenship, Tracy Riggins

**Affiliations:** ^1^Neuroscience and Cognitive Science Program, University of Maryland, College ParkMD, USA; ^2^Department of Psychology, University of Maryland, College ParkMD, USA

**Keywords:** memory, executive function, unitization, binding, development

## Abstract

Previous research has documented an association between executive functioning (EF) and memory for bound details. However, it is unknown if this relation varies as a function of the type of bound information (i.e., unitized versus non-unitized) and whether this association changes as a function of age during childhood, when both EF and memory undergo rapid development. The current study sought to address these gaps by examining whether relations between parent-reported EF differed for unitized versus non-unitized memory representations and if these relations differed between children who were 4, 6, or 8 years of age. Results revealed that EF was selectively associated with non-unitized associative memory in 8-year-old children; no significant relations between EF and either memory condition were evident in 4- or 6-year-olds. These results suggest relations between EF and memory may be specific to non-unitized representations and that this association may emerge across childhood as both EF and memory abilities develop.

## Introduction

Part of what transforms memories into meaningful personal experiences is the ability to bind a variety of details into cohesive memory wholes: for instance, not only remembering *who* you met, but also, *what* you discussed, *where* you were, and *when* the meeting took place. Given the importance of remembering such details, a great deal of research in adults and children has focused on how these separate elements are represented and bound together in order to create rich, detailed memories for past experiences (e.g., Spencer and Raz, 1994; Glisky et al., 1995; Chalfonte and Johnson, 1996; Cycowicz et al., 2001; Mitchell et al., 2004; Sluzenski et al., 2006; Lloyd et al., 2009; Picard et al., 2012). Research on this topic has typically used laboratory-based paradigms to examine binding of two separate items together (e.g., two individual pictures), binding of an item with its source (e.g., the individual from whom a fact was learned), binding an item with a feature (e.g., the color or shape of the object), or binding an item with a context (e.g., an item in a particular spatial or temporal location). Although differences may exist in the processes (e.g., sensory, neurofunctional) engaged during these tasks, what they all have in common is the joining of separate pieces of information to create a bound representation.

### Relations between Memory and EF

Bound memory representations have been shown to differ from unbound memory representations (i.e., memory for individual items) in multiple ways (Spencer and Raz, 1995; Chalfonte and Johnson, 1996; Dobbins et al., 2002; Drummey and Newcombe, 2002; Siedlecki et al., 2005; for review see Old and Naveh-Benjamin, 2008). Of greatest relevance to the present report, bound and unbound memories have been suggested to rely differentially on executive functions (EFs), domain-general cognitive processes involved in the planning, organization, and execution of complex goal-directed behaviors. EFs are proposed to support recollection of bound representations by guiding systematic searches for memory traces, maintaining information in working memory, and inhibiting irrelevant information (Shimamura, 2002; Buckner, 2004; Blumenfeld and Ranganath, 2007; Raj and Bell, 2010; Ranganath, 2010). Based on this premise, EFs may play a greater role when more than one item or trace must be retrieved, evaluated, and decided upon in comparison to recall of individual items (Mitchell et al., 2000; Dobbins et al., 2002). Although some studies have demonstrated associations between EF and item memory (e.g., Spencer and Raz, 1994), given our hypotheses regarding the involvement of EFs in bound representations, we will focus our discussion on relevant studies from the literature.

The hypothesis that EF is related to memory for bound representations has been supported by multiple lines of research. Adult neuroimaging investigations suggest the encoding and recall of bound information preferentially activates prefrontal cortices that subserve EF (Cabeza et al., 2000; Dobbins et al., 2002; Mitchell et al., 2004; Blumenfeld and Ranganath, 2007). For example, retrieval of episodic details is consistently associated with prefrontal activation in comparison to retrieval of single items (for review see Bunge et al., 2004; Murray and Ranganath, 2007; Preston and Eichenbaum, 2013). Similarly, the association between frontal lobe function and memory for bound representations has been demonstrated in aging populations and patients with prefrontal lesions (Janowsky et al., 1989; Craik et al., 1990; Spencer and Raz, 1994; Glisky et al., 1995, 2001; Siedlecki et al., 2005; Smith et al., 2011; Giovanello and Schacter, 2012).

The association between EF and memory for bound representations also exists in childhood (Cycowicz et al., 2001; Picard et al., 2012; Earhart and Roberts, 2014; Rajan et al., 2014; for review see Raj and Bell, 2010), although exceptions exist (e.g., Drummey and Newcombe, 2002). For example, EFs (inhibition, working memory, and shifting) have been shown to be related to 3- to 6-year-old children’s ability to recall the source from whom facts were learned, but were not related to recognition of facts alone (Rajan et al., 2014). Similarly, Picard et al. (2012) used a novel event memory paradigm (the House Task) in 4- to 16-year-old children and showed that measures of EF (working memory, inhibition, shifting) were related to memory for spatial (location) and temporal (time of day) details, but not individual items. Cycowicz et al. (2001) used an item-color memory paradigm with 7- to 9-year-old children where memory for color was correlated with EF (working memory, inhibitory control), but item recognition was not. Moreover, similar to the adult literature, retrieval of episodic details in children is associated with prefrontal activation (Ofen et al., 2007; Ghetti and Bunge, 2012) and greater connectivity between the PFC and the MTL is associated with improved memory performance (Menon et al., 2005; Ofen et al., 2012).

### Open Questions Regarding the Relations between Memory and EF

Thus, the developmental literature suggests EFs (behaviorally and neurally, by way of PFC activation) are often associated with the recall of bound representations; however, the nature of these associations remains unclear. First, it is unknown how specific the relations between EF and bound memory representations are: do these relations exist for all types of bound information or only some? This question arises in light of research demonstrating at least two ways to bind information: via unitization, in which the detail becomes a feature of the item or via non-unitization, in which the detail remains distinct from the item (e.g., Diana et al., 2010, see also Moscovitch, 1992). For example, when asked to remember the item ‘dog’ and the color ‘green,’ one can unitize these two entities making the color a *feature* of the dog, creating a single unitized representation of a ‘green dog.’ In contrast, one could bind ‘dog’ and ‘green’ by associating the dog with the green dollar bill it ate. In this case, ‘green’ is no longer a feature of the dog, but is instead a detail that is bound with the item ‘dog.’ In both conditions individuals are required to bind ‘green’ and ‘dog,’ but it is proposed that the fundamental binding mechanism differs between unitized versus non-unitized conditions, resulting in different types of representations (Diana et al., 2010; Bastin et al., 2013; see also Moscovitch, 1992). Thus, it may be the case that unitized binding may only require the search and retrieval of a single representation and therefore be more similar to item memory and rely less on EFs (Bader et al., 2014). For example, in adults, Bader et al. (2014) examined neural activation during unitized and non-unitized encoding of novel word pairs and found that words unitized into a novel conceptual unit (e.g., milk taxi = a milk delivery service) demonstrated reduced involvement of the network of brain regions implicated in recollection – providing evidence that unitized binding of words relies more heavily on familiarity (versus recollection) processes and is more akin to item memory. Similarly, in adults, Piekema et al. (2010) reported robust PFC activation during inter-item binding (non-unitized; other item) while intrinsic intra-item (unitized; color) did not show any differential activation. Unfortunately, other investigations using these operationalizations of binding have limited analyses to medial temporal lobe regions, largely failing to discuss activation in prefrontal cortices (Diana et al., 2007, 2010).

A second open question is whether relations between EF and bound memory representations change with age. Ample evidence exists indicating that both children’s EF abilities and their ability to remember bound details undergo dramatic developmental changes during childhood. Specifically, developmental studies of EF consistently report age-related improvements across a variety of subdomains (e.g., inhibition, shifting, working memory), from infancy through young adulthood (e.g., Anderson, 2002; for review, see Best et al., 2009). There is less consistency, however, regarding which subdomains are specifically related to memory (Ruffman et al., 2001; Picard et al., 2012; cf. Earhart and Roberts, 2014; Rajan et al., 2014). These conflicting results may, in part, be explained by the argument that the organization of EFs change over development, from relatively unitary to more functionally distinct. For example, Wiebe et al. (2008) argue EFs comprise a single factor through age 6, whereas evidence from Lehto et al. (2003) suggests that by age 8, a three-component model is most appropriate (Miyake et al., 2000). Relatedly, it has been proposed that laboratory-based neuropsychological tasks (e.g., Stroop, Digit Span), which are often designed to measure a single subdomain, may only partially reflect ‘real-world’ executive competencies (Vriezen and Pigott, 2002; Barkley and Murphy, 2011). A recent meta-analysis revealed that performance on laboratory-based tasks is only moderately correlated with parent-report measures of EF (Toplak et al., 2013). This moderate effect size suggests that parent-report measures may provide unique and complementary indices of EF that may not be captured in neuropsychological behavioral tasks (Isquith et al., 2004; Toplak et al., 2013; Nguyen et al., 2014). Given EFs show substantial development throughout childhood, it may be expected that as these abilities and their neural substrates mature, they will play different roles in memory processes. For instance, Glisky and Kong (2008) report EF is associated with source memory performance in older adults, but not young adults – suggesting the association between these two cognitive capacities may undergo developmental change (see Spencer and Raz, 1994 for age-related changes in the association between EF and memory for facts and contextual details). Similarly, children’s memory ability also shows substantial improvement during early childhood (Bauer and Fivush, 2013). In fact, multiple studies have identified the transition from early to middle childhood as being a time of rapid change in children’s ability to recall contextual or bound details (Drummey and Newcombe, 2002; Sluzenski et al., 2006; Riggins, 2014).

### Current Investigation

Given contemporaneous development of EF and memory during childhood, albeit with potentially differing rates and trajectories, it is reasonable to suggest age-related differences in how these cognitive processes may support each other throughout development. This hypothesis is supported by neuroimaging evidence (Ofen et al., 2007; Ghetti and Bunge, 2012, described above), which suggests age-related improvements in episodic memory may be accounted for, in part, by changes in prefrontal connectivity. Although research has examined relations between EF and memory in childhood (e.g., Rajan et al., 2014), to date, no study has explicitly examined age related differences in the association between EF and memory performance.

The goal of the present study was to explore the nature of the relation between EF and memory. First, the question of how EF may support distinct binding processes during childhood was examined by comparing the relation between EF and unitized versus non-unitized representations. Second, developmental changes in the relation between EF and memory were examined by comparing correlations between EF and memory in 4-, 6-, or 8-year-old children. Memory stimuli were adapted from a previous investigation in adults (Diana et al., 2010) to yield unitized and non-unitized representations. Keeping the extant literature in mind, we employed a parent-report measure of EF [Behavior Rating Inventory of Executive Function (BRIEF)] that includes ecologically valid, age-appropriate global measures (capturing EF as a single factor) as well as individual subdomains (providing indices of a multi-component model of EF, Isquith et al., 2004). We hypothesized that EF would be specifically related to non-unitized memory performance due to a reliance on a systematic search and decision on multiple memory traces. Given that EF and memory systems undergo different developmental trajectories, analyses investigating age-related changes in these associations were exploratory.

## Materials and Methods

### Participants

A total of 102 children (60 female) participated in the study. Four participants (two 4-year-old males, one 4-year-old female, and one 6-year-old female) were excluded from analyses due to failure to follow instructions. The remaining 98 children comprised three age groups of interest: 4-year-olds (*n* = 41, female = 26, *M* = 4.59 years, SD = 0.29, range = 4.00–4.94), 6-year-olds (*n* = 33, female = 15, *M* = 6.58 years, SD = 0.27, range = 6.03–7.00), and 8-year-olds (*n* = 24, female = 17, *M* = 8.52 years, SD = 0.23, range = 8.05–8.96). Of the 57 families who chose to report household income (17 4-year-olds, 23 6-year-olds, 17 8-year-olds), the median family income was >$75,000 per year. Of the 95 families who reported parent education, 89 families (93.68%) listed at least one parent with a 4-year college degree. Children were typically-developing; exclusion criteria included: colorblindness, diagnosed or suspected psychopathology, developmental disabilities, language or learning difficulties, prematurity, previous head injury or unconsciousness, or serious behavioral problems.

### Stimuli

Training stimuli were used to ensure understanding of the task and included a small plastic mouse, a small plastic bird, a small wooden stop sign, a paper dollar bill, and two crayons (one red, one green). Stimuli consisted of 72 black and white images (12 practice and 60 test items) presented on 3″ × 4″ notecards. Images were chosen from the Snodgrass and Vanderwart (1980) line drawings with additional images selected to match in visual complexity. Each image was paired with a one-sentence story for each condition (described below). Stories were adapted from a previous study with adults (Diana et al., 2010) to be age-appropriate. All children saw the same 60 test stimuli^1^; however, the condition in which the image appeared was counterbalanced between children. For instance, child 1 viewed set A, which included items 1–30 in the Non-Unitized condition and items 31–60 in the Unitized condition, whereas child 2 viewed set B, which included items 1–30 in the Unitized condition and items 31–60 in the Non-Unitized condition (see below for additional procedural details; see Supplementary Material for full stimulus list). Stimuli included in the present investigation were chosen based on a pilot study that was conducted to acquire a stimulus set which equated performance on both memory conditions in an independent sample of 8 year-olds. This approach, used by Diana et al. (2010) to equate performance in adults, was based on assumptions drawn from previous research which suggest by 8 years of age, children would have mature levels of unitized and non-unitized binding capabilities (Drummey and Newcombe, 2002; Sluzenski et al., 2006; Riggins, 2014).

### Procedure

Participants completed the experiment in the lab or in a research testing room at an on-campus preschool. All parents provided informed consent. Eight-year-old participants also provided written informed assent. Methods were approved by the Institutional Review Board prior to the start of testing.

#### Memory Paradigm (Adapted from Diana et al., 2010)

**FIGURE 1:**
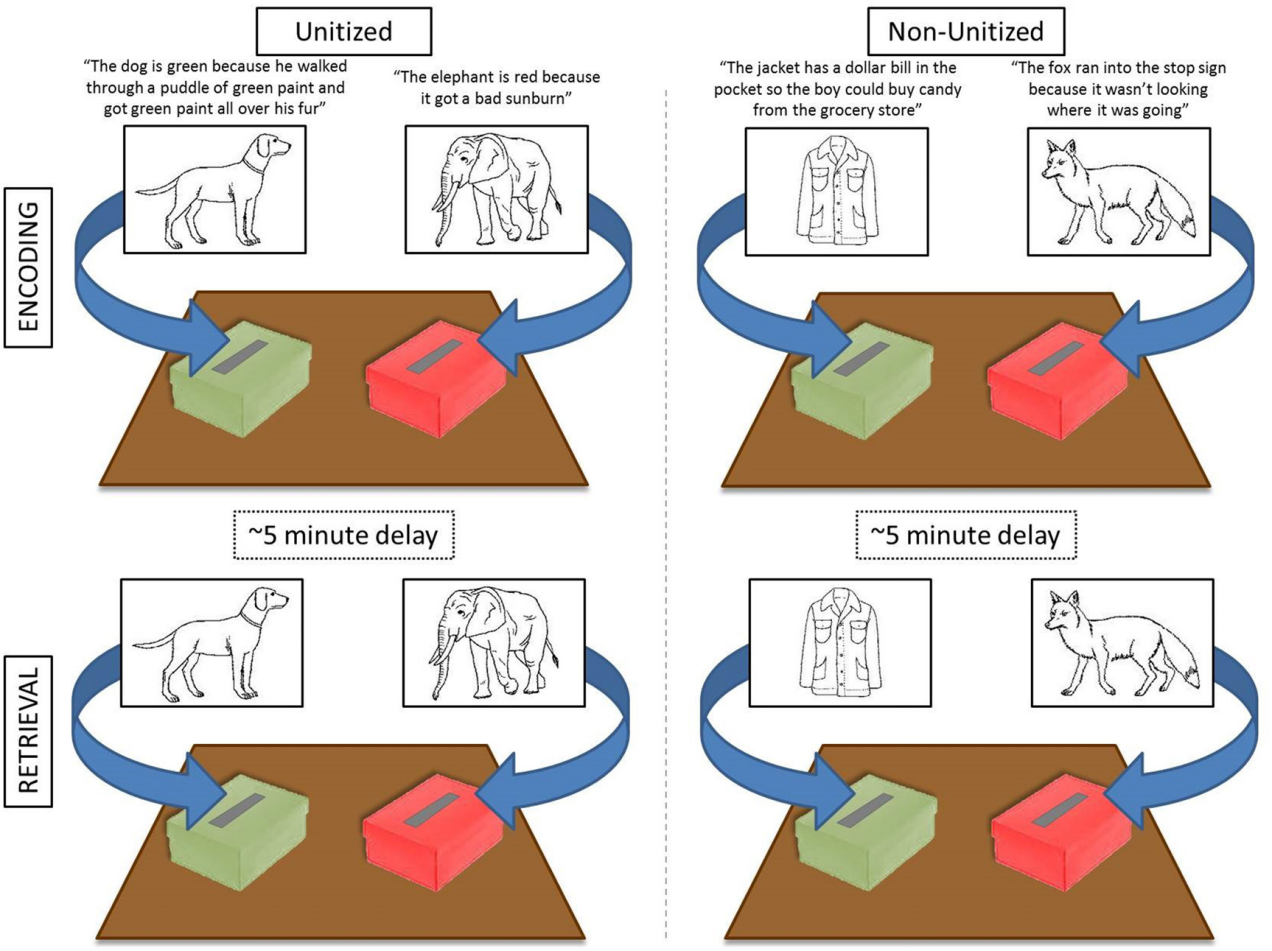
**Schematic of experimental design.** Two colored boxes were placed on the table in front of the child. During each encoding block (i.e., Unitized or Non-Unitized; top panels) children were presented with 30 stimuli, one at a time, and instructed to place the card into the appropriate box based on the story. At retrieval, the same images were randomly presented without the accompanying story and children were instructed to re-sort the items into the appropriately colored box (bottom panels). Each encoding block was followed by a brief distraction task and the retrieval portion for that block. Block presentation was randomized between participants. Stimuli from Snodgrass and Vanderwart (1980).

Each child participated in two blocked conditions: Unitized and Non-Unitized (see **Figure 1**). Both conditions required memory of the association between an image and a color. In the Unitized Condition, the color (red or green) was intended to become a feature of the image (e.g., “*The elephant is red because it got a bad sunburn*”/“*The dog is green because he walked through a puddle of green paint and got green paint all over his fur*”). In contrast, in the Non-Unitized Condition color was bound to images via association with a colored object (i.e., a stop sign or a dollar bill; e.g., “*The fox ran into the stop sign because it wasn’t looking where it was going”*/“*The jacket has a dollar bill in the pocket so the boy could buy candy from the grocery store”*).

Prior to completing the task, training and practice trials were administered to ensure task understanding. Children completed two training trials per condition. For the Unitized Condition, children were asked to discriminate between a red and a green crayon to screen for color-blindness and ensure appropriate color knowledge to complete the sorting task. No children failed this discrimination task. Once children correctly identified the crayons, they were asked to color two line drawings based on a story told by the experimenter (i.e., “*The dog is green because he walked through a puddle of green paint and got green paint all over his fur”* and “*The tiger is red because it ate so many red popsicles it turned red just like a popsicle*”). During Non-Unitized practice trials children were asked to identify the name, use, and color of a miniature toy stop sign and a vivid-green dollar bill. The experimenter then acted out a story using toys and the child was asked to imitate the experimenter’s stories (i.e., For the story “*The mouse ran up the stop sign to get away from the cat,”* the experimenter would show a miniature toy mouse running up a miniature toy stop sign). These training trials were designed to facilitate imagining stories in unitized or non-unitized ways. Following training, children completed six practice trials per condition. Practice trials were presented with the same instructions and structure as the test trials (explained below). Feedback was provided during practice trails if items were incorrectly sorted at encoding or retrieval.

At encoding, children were shown black and white images presented on 3″ × 4″ notecards while the experimenter read the associated Unitized or Non-Unitized story. Children were instructed to imagine the image as it was described in the story and sort the card into the appropriately colored box, red or green, which were placed on the table in front of them. This adaptation was developmentally appropriate and (1) made the task more engaging to our younger participants and (2) ensured adequate attention to the stories and encoding of the relevant details. For stories about green items (Unitized Condition) or items paired with a dollar bill (Non-Unitized Condition) children were instructed to put the card into the green box. For stories about red items (Unitized Condition) or items paired with a stop sign (Non-Unitized Condition) children were instructed to put the card into the red box. Children were corrected if they sorted an image incorrectly. If the child identified their error spontaneously and/or could provide verbal recollection of the reason their original response was wrong, the sentence was not repeated. If the child could not recall the story, the sentence was repeated and the child resorted the card into the correct box^2^. Children were told to remember which box they sorted the item into because they would be asked about it later.

At retrieval, children were randomly presented each of the previously encoded pictures and asked to re-sort the item into the appropriate box. During retrieval, the relative positions of the boxes were switched (i.e., if the red box was originally on the child’s left, it was moved to the right) to eliminate any spatial-cue related confounds. Children were not corrected if items were incorrectly sorted during retrieval. Breaks were given between encoding and retrieval (∼5 min) and between condition blocks (∼10 min). The dependent measure was the percentage of items (out of 30) correctly sorted at retrieval.

Each condition consisted of 30 test trials: 15 red/15 green for the Unitized Condition and 15 stop sign/15 dollar bill for the Non-Unitized Condition. Condition order was randomly assigned between participants and stimuli were randomized within participants with the constraint that no more than two trials of a single color/association were presented in a row.

#### Executive Function

The BRIEF (Gioia et al., 2000), a parent-report questionnaire, was used to index EF. The advantages of this questionnaire are that it is moderately related to laboratory-based tasks (Toplak et al., 2013) yet it also provides additional information about EF “in the real world” and that it yields standardized scores that can be compared across wide age ranges with differing abilities (i.e., 4- to 8-year-olds). The preschool version (ages 2–5 years, BRIEF-P) has 63 items and five subscales (Inhibit, Shift, Emotional Control, Working Memory, and Plan/Organize; Gioia et al., 2003). The standard version (ages 5 years and up, BRIEF) has 86 items and eight subscales (Inhibit, Shift, Emotional Control, Initiate, Working Memory, Plan/Organize, Organization of Materials, and Monitor; Gioia et al., 2000). Both the BRIEF-P and BRIEF provide raw and scaled T-scores for each subscale and a Global Executive Composite (GEC) score. T-scores were assigned according to the BRIEF scoring manual, where values are calculated based on age and gender, as these factors were found to influence EF. Larger scores on the BRIEF indicate greater executive dysfunction.

While children completed the behavioral session in the laboratory, parents/guardians filled out a number of questionnaires, including the BRIEF Standard or Preschool version^2^. Parents of 4-year-old children completed the BRIEF-P^3^, whereas 6- and 8-year-old children completed the BRIEF in order to capture developmentally appropriate EFs. 25 participants were excluded from questionnaire analyses for the following reasons: parents of children tested at the preschool did not complete the questionnaire (13 4-year-olds, 2 6-year-olds), parent failure to answer all items necessary for GEC calculations (two 6-year-olds; one 8-year-old), parent completing the BRIEF instead of the BRIEF-P (one 4-year-old), and failure to complete any of the BRIEF or BRIEF- P (one 4-year-old; four 6-year-olds; one 8-year-old). These exclusions resulted in slight sample size variations in subsequent analyses.

### Analytic Approach

The two primary goals of this investigation were to: (1) determine the specificity of the relations between parent-report EF and memory condition and (2) explore if this relation differs as a function of age. As such, correlations were used to examine relations between EF and each condition in each age group. Given the fundamental differences in calculation of raw scores between the BRIEF and BRIEF-P, standardized T-scores were used to examine differences in EF between age groups. In order to examine relations between EF and memory, separate partial correlation analyses were conducted between measures of memory performance and standard (T) scores of the GEC for each condition (Unitized, Non-Unitized) and age group (4, 6, 8); within each age group, age was entered as a covariate to ensure that developmental differences within an age group would not account for the observed within-group correlations. When significant associations were observed, correlation values were compared between conditions and between age groups to determine whether they were significantly larger by using Fisher’s r-to-z transformations and one-tailed tests. When significant associations were found between memory and the GEC, follow-up analyses were conducted comparing scores on the subscale measures and memory to identify if these associations were driven by specific EF components. Because the development of EFs has been linked to factors such as parent education (Ardila et al., 2005) and family income (Hackman et al., 2015), follow-up analyses were run controlling for parent education or family income and age.

## Results

### Memory

**FIGURE 2:**
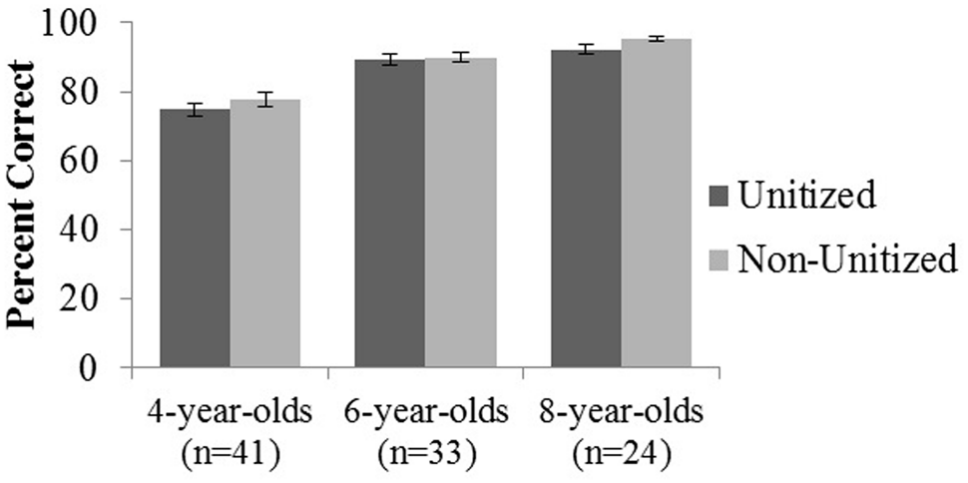
**Memory performance for 4-, 6-, and 8-year-old children on unitized and non-unitized memory conditions.** Error bars represent standard error of the mean.

A 3 Age Group (4, 6, 8 years) × 2 Condition (Unitized, Non-Unitized) mixed analysis of variance (ANOVA) revealed a main effect of age, *F*(2,95) = 51.947, *p* < 0.001, η^2^ = 0.52 (**Figure 2**). Pairwise comparisons revealed that 4-year-olds performed significantly below both 6- and 8-year-olds, but there was no significant difference in performance between 6- and 8- year-old (**Table 1**). Across age groups, performance on the Non-Unitized Condition (*M* = 87.53 ± 9.95) was nominally greater than the Unitized Condition (*M* = 85.33 ± 9.49), but this failed to meet traditional thresholds for statistical significance, *p* = 0.08. There was no Age × Condition interaction, *p* = 0.68. A similar pattern of findings was observed in the subset of children whose parents filled out the EF questionnaires.

**Table 1:** Descriptive statistics for memory performance (percent correct) by age and trial type.

Group	Mean (%)	SD	Range
Four-year-olds (*n* = 41)			
Unitized			
Green Correct	74.96	17.56	13–100
Red Correct	74.47	13.66	47–100
Non-Unitized			
Stop Sign Correct	73.82	16.63	26.67–100
Dollar Bill Correct	81.79	13.34	53.33–100
Six-year-olds (*n* = 33)			
Unitized			
Green Correct	89.70	8.83	67–100
Red Correct	88.69	10.47	60–100
Non-Unitized			
Stop Sign Correct	87.47	10.90	46.67–100
Dollar Bill Correct	92.12	8.89	73.33–100
Eight-year-olds (*n* = 24)			
Unitized			
Green Correct	91.94	7.61	80–100
Red Correct	92.22	7.53	73–100
Non-Unitized			
Stop Sign Correct	93.89	6.79	80–100
Dollar Bill Correct	96.39	4.39	86.67–100

### Executive Function

A one-way ANOVA examining differences between Age Groups for the BRIEF GEC score revealed similar standardized T-scores between 4-year-olds (*M* = 46.00 ± 7.35, range = 35–64), 6-year-olds (*M* = 50.40 ± 7.82, range = 36–67), and 8-year-olds (*M* = 46.23 ± 6.43, range = 37–58), *p* = 0.06, suggesting similar EF abilities between groups, after accounting for the effects of age.

### Relations between Memory and Executive Function

No significant relations were observed between EF and either memory condition for 4- or 6-year-old children. However, results revealed that, for 8-year-olds, Non-Unitized associative memory was related to parent-reported EF ability (**Table 2**), but Unitized memory was not. Comparisons of the correlation values in the 8-year-old group revealed that the correlation between Non-Unitized memory and EF was significantly greater than the correlation between Unitized memory and EF, and that the relation between Non-Unitized memory and EF was significantly greater in 8-year-olds compared to that with either 6- or 4-year-olds (see **Table 2**). These associations remained unchanged when controlling for household income and parent education. All 4-year-olds reported a household income > $75,000 and thus these effects could not be tested. Partial correlations between GEC and memory demonstrated the same pattern of results after controlling for age and household income in 6-year-olds (Unitized: *r* = 0.149, *p* = 0.56, Non-Unitized: *r* = 0.026, *p* = 0.92) and 8-year-olds (Unitized: *r* = -0.150, *p* = 0.61; Non-Unitized: *r* = -0.695, *p* = 0.006). Similarly, trends remained unchanged when controlling for age and parent education for 4-year-olds (Unitized: *r* = -0.320, *p* = 0.12; Non-Unitized: *r* = 0.142, *p* = 0.517), 6-year-olds (Unitized: *r* = 0.132, *p* = 0.559; Non-Unitized: *r* = 0.123, *p* = 0.586), and 8-year-olds (Unitized: *r* = -0.142, *p* = 0.550, Non-Unitized: *r* = -0.579, *p* = 0.007). Although these analyses suggest the present results cannot be attributed to confounding factors associated with parent education or household income, they should be interpreted with caution, as there was limited variability in the sample, with most children coming from upper middle class families with at least one parent with a 4-year college degree.

**Table 2:** Partial correlation of global executive function and condition, controlling for age.

Age	Condition				
	Unitized		Non-Unitized		
	*r^∗^*	*p*	*r^∗^*	*p*	Condition comparison^†^
4 (*n* = 26)	-0.35_a_	0.08	0.10_a_	0.64	*ns*
6 (*n* = 25)	-0.02_a_	0.94	-0.03_a_	0.90	*ns*
8 (*n* = 22)	-0.14_a_	0.55	-0.63_b_	0.002	*z* = 1.85, *p* = 0.04

Higher scores on the BRIEF indicate greater difficulties with executive function; therefore, the negative correlations presented here indicate better EF is associated with better memory performance. Correlation values with different letters are significantly different from each other. *p < 0.05, two-tailed; ^†^*p* < 0.05, one-tailed.

Finally, we examined whether specific domains of EF contributed to the observed association between Non-Unitized memory and EF in 8-year-olds. Results indicated that the association between Non-Unitized memory and EF was not driven by any one subscale of EF, as each of the following subscales were related to Non-Unitized memory in 8-year-olds (Shifting *p* = 0.014; Planning and Organizing, *p* = 0.005; Initiating, *p* = 0.029; Organization, *p* = 0.003; Monitoring, *p* = 0.049) and only three subscales were not related (Inhibition, Emotional Control, and Working Memory, *p*s > 0.10).

## Discussion

This is the first study, to our knowledge, to investigate the specificity of relations between memory for bound associations and EF as well as whether these associations vary across childhood. Results indicated that global, parent-reported EF is selectively related to Non-Unitized memory, but not Unitized memory, in 8-year-old children only. This pattern of results suggests that the relation between EF and memory is specific as there was not a reliable difference in accuracy between these memory conditions, yet only performance on the Non-Unitized Condition was significantly related to EF. In addition, these results suggest that the relation between EF and memory may vary as a function of age, as the correlation between EF and Non-Unitized memory in 8-year-olds was significantly greater than the correlation in either 4- or 6-year-old children. Although memory performance was worse in 4-year-old children, there were no differences in memory between 6- and 8-year-old children. Thus, despite similar levels of performance and similar variability in 6- and 8-year-olds, EF was selectively related to memory in 8-year-old children, with greater EF (lower BRIEF scores) associated with better Non-Unitized memory performance.

The specificity of the relation between Non-Unitized binding and EF is consistent with the theoretical perspective that EF is more engaged during the search, retrieval, and maintenance of multiple representations. Specifically, EFs may be supporting the recollection of multiple representations by guiding systematic searches for memory traces, maintaining information in working memory, and inhibiting irrelevant information (for discussion in adults, see Shimamura, 2002; Buckner, 2004; Blumenfeld and Ranganath, 2007; Ranganath, 2010; for discussion in children, see Raj and Bell, 2010). However, this finding was only observed in 8-year-old children. This is somewhat surprising because, after correcting for age (by using standardized scores), levels of EF were similar between 6- and 8-year-old age groups and there was no difference in memory performance. What may be driving this change? It may be the case that developmental changes in the organization or structure of EF may be occurring during this period. It has been proposed that between 6 and 8 years, EF transitions from a unitary factor to a three-component process (Miyake et al., 2000; Lehto et al., 2003; Wiebe et al., 2008). These changes may be tightly coupled to developmental changes in neural organization. There is ample evidence that childhood is marked by a relative transition toward greater functional specialization – specifically, Fair et al. (2009) demonstrated that between 7 and 31 years, prefrontal cortices segregate into functionally distinct networks. Further evidence from studies in school-aged children indicate age-related changes in prefrontal involvement during memory encoding (Menon et al., 2005; Chiu et al., 2006; Ofen et al., 2007; Ghetti et al., 2010) and retrieval (Ofen et al., 2012; DeMaster and Ghetti, 2013; for review, see Ghetti and Bunge, 2012). Taken together with evidence from Anderson (2002) that EFs undergo rapid development between 7 and 9 years, these behavioral and neural changes may underlie the change in association between memory performance and EF between 6 and 8 years.

The lack of association between Unitized memory and EF may appear to differ from previous literature. For example, previous studies have reported that recall of item color is associated with EF in children (Cycowicz et al., 2001 and adults (Spencer and Raz, 1994). However, these previous studies did not specifically address whether the memory representation was unitized or not. It could be the case that participants in these studies encoded details in a non-unitized fashion. In addition, these studies differed in the methods for measuring EF, namely neuropsychological tests of EF subdomains, which may contribute to the difference in results. Further research is needed to determine the source of this apparent difference. In contrast, the current study replicates previous literature examining memory development (without regard to EF) by demonstrating age-related improvement in memory for bound details between 4 and 6 years (Drummey and Newcombe, 2002; Sluzenski et al., 2006; Bauer et al., 2012; Riggins, 2014). Interestingly, memory improvements were evident for both Unitized and Non-Unitized conditions suggesting the processes supporting memory for bound details, although distinct, may show similar development between 4 and 6 years. However, there are currently no data to speak to this issue directly and thus this issue also awaits future investigation.

Performance on the two memory conditions was similar in all age groups. This is in contrast to evidence from dual process theories of memory, which suggest that unitized binding may be supported by familiarity, which matures earlier than recollection, on which non-unitized binding is thought to rely (Ghetti and Angelini, 2008; Diana et al., 2011). Relatedly, behavioral studies have demonstrated different developmental trajectories for item memory and episodic memory (e.g., Drummey and Newcombe, 2002; Riggins, 2014). Therefore, if the assumption that unitized binding is akin to item memory is true, one may expect to see differences in age-related improvements between the two groups. However, no Age × Condition interactions were observed in the present study. A few factors of our experimental design may contribute to these results. First, items included in the present investigation were chosen to equate performance on both memory conditions in 8 year-olds. It is possible that these stimuli may have minimized the difference between conditions in the other age groups as well. Second, the Unitized sentences in the current investigation may have engaged more “shallow” encoding processes in comparison to the Non-Unitized pairings. For example, many Unitized sentences relied on somewhat arbitrary associations such as “The castle is red because it belongs to a king who loves the color red” while Non-Unitized sentences may have created a stronger narrative which supported deeper encoding: “The castle has a stop sign on the road in front of it so people can stop and take pictures of the beautiful building.” The shallowness of the associations may have influenced the depth of encoding processes, artificially deflating Unitized performance in comparison to Non-Unitized. As such, future investigations should experimentally control for the arbitrariness or meaningfulness of associations across conditions. Second, evidence suggests the success of forming bound representations (Unitized and Non-Unitized) may be moderated by the ease of visualizing the association (Bastin et al., 2013). Since there was no check in the current investigation to ensure children were indeed imagining the described associations, performance on both conditions may have been compromised. This effect may have been rescued in the Non-Unitized Condition due to greater reliance on EF supporting a more systematic weighting of memory traces, resulting in more accurate completion of the task.

There are several additional questions that should be addressed by future research. First, the present study used parent-reported EF in order to index real-world EF and avoid the limitations of laboratory-based tasks. Although our use of parent-reported EF adds richness to the extant literature by examining the association between non-task-based measures of EF and memory performance, there are some limitations to parent-report measures that should be noted. First, parent-report and task-based measures of EF are only moderately correlated (Toplak et al., 2013), leaving open questions regarding the underlying nature of each measure. Additionally, as with all questionnaire measures, it is difficult to standardize between-subject reporting behaviors, which may have resulted in some parents over-estimating the frequency of problem behaviors (Vriezen and Pigott, 2002). Because the current approach differed from previous studies, it remains unclear why findings in the present report with the Unitized memory condition differed from previous studies (Spencer and Raz, 1994; Cycowicz et al., 2001; cf. Drummey and Newcombe, 2002; Guillery-Girard et al., 2013). Thus, in future research it would be beneficial to employ multiple methods that allow the systematic comparison of observational, parent-report, and neuropsychological measures of EF. Additionally, future investigations could extend the current findings by examining the relation between EF and other conceptualizations of binding processes. For example, studies should compare Non-Unitized binding of different details (e.g., temporal binding, spatial binding, source binding, paired associations, etc.; see Picard et al., 2012; Guillery-Girard et al., 2013) to elucidate the specificity (or generality) of EFs on developing binding processes as well as associations with simple item memory. Third, neuroimaging investigations would be well-suited to explore the neural mechanisms underlying the current findings. For example, fMRI studies could identify whether retrieval of non-unitized associations in 8 year-olds engaged prefrontal/EF regions to a greater extent than unitized binding. Finally, these studies could examine at what point in the memory process EFs exert their greatest influence. For instance, it remains inconclusive whether mature EFs allow for strategic encoding and/or if they simply support the systematic search for representations during retrieval (see Murray and Ranganath, 2007; Güler and Thomas, 2013).

In sum, the goal of this research was to explore the nature of the relation between EF and unitized and non-unitized memory representations in childhood. Results suggest EF plays a greater role in memory of non-unitized representations compared to unitized representations and that this association emerges across childhood. Together these findings suggest caution in extrapolating findings about the relation between memory and EF in certain age groups (e.g., middle childhood) to other developmental periods (e.g., early childhood) as well as findings between some memory tasks (e.g., non-unitized tasks) to other tasks (e.g., unitized tasks). Consideration of the bidirectional influences of the development of EF and memory may help elucidate the mechanisms driving age-related changes observed in both cognitive abilities during the childhood years.

## Author Contributions

SB: data collection, analysis, writing; TR: study design, analysis, writing.

## Conflict of Interest Statement

The authors declare that the research was conducted in the absence of any commercial or financial relationships that could be construed as a potential conflict of interest.

## Footnotes

1 Due to experimenter error, three children viewed fewer stimuli at encoding (57–59). Percent correct for these children was calculated using the appropriate denominator.

2 Four-year-olds mis-sorted cards more frequently than 6- or 8-year-olds [*F*(2,94) = 6.692, *p* = 0.002; 4-year-olds *M* = 1.37, SD = 1.84, 6-year-olds *M* = 0.53, SD = 0.95, 8-year-olds *M* = 0.21, SD = 0.42 for Unitized; *F*(2,94) = 8.782, *p* < 0.001; 4-year-olds *M* = 1.61, SD = 1.88; 6-year-olds *M* = 0.66, SD = 1.07; 8-year-olds *M* = 0.21, SD = 0.42); however, there was no difference between the groups in number of items repeated for either condition (*p* = 0.65 Unitized; *p* = 0.135 Non-Unitized).
