# Supplementary material for: Developmental differences in relations between parent-reported executive function and unitized and non-unitized memory representations during childhood
Source: Front Psychol. 2015 Aug 19;6:1214. doi: 10.3389/fpsyg.2015.01214 (PMC4541290; doi:10.3389/fpsyg.2015.01214)
Supplement: Supplementary file 1 [file Stimuli.DOCX]

***Supplementary Material***

**Developmental differences in relations between parent-reported executive function and unitized and non-unitized memory representations during childhood**

**Sarah L Blankenship^1^, Tracy Riggins*^2^**

^1^Neuroscience and Cognitive Science Program, University of Maryland, College Park, MD, USA.

^2^Department of Psychology, University of Maryland, College Park, MD, USA.

*Correspondence: Dr. Tracy Riggins, Department of Psychology, University of Maryland,

College Park, MD, 20742, USA.

riggins@umd.edu

Appendix

| **Stimulus** | **Set A** | **Condition** | **Set B** | **Condition** |
| --- | --- | --- | --- | --- |
| 1. **banana** | The banana is green because it was just picked off the tree and is not ready to eat yet. | Unitized | The banana goes with the dollar bill because the clown rolled it up to look like a banana as a joke. | Non-Unitized |
| 1. **bed** | The bed is green because it is for a green dragon to sleep in. | Unitized | The bed had a dollar bill under it because that's where the robber hid the stolen money. | Non-Unitized |
| 1. **block** | The block is green because it is part of a Lego set that makes a park. | Unitized | The block goes with a dollar bill because the little boy found it under the chair with his missing block. | Non-Unitized |
| 1. **calf** | The calf is green because it rolled in the fresh grass and got stained. | Unitized | The calf is eating dollar bills because it got confused and thought it was a piece of grass. | Non-Unitized |
| 1. **eye** | The eye is green because it belongs to a cat whose eyes are green. | Unitized | The eye goes with the dollar bill because there is a person's face with eyes on it. | Non-Unitized |
| 1. **ham** | The ham is green because it is in a new Dr. Seuss book called green ham and eggs. | Unitized | The ham had a dollar bill stuck to it because the dollar fell out of the chef's pocket while he was cutting the ham. | Non-Unitized |
| 1. **jacket** | The jacket is green because the mascot is a leprechaun and green is the team color. | Unitized | The jacket has a dollar bill in the pocket so the boy could buy candy from the grocery store. | Non-Unitized |
| 1. **rabbit** | The rabbit is green because it ate so much lettuce it started to look green, like the lettuce. | Unitized | The rabbit goes with a dollar bill because the parents bought it from a farmer for a dollar. | Non-Unitized |
| 1. **rug** | The rug is green because it goes outside and is supposed to look like grass. | Unitized | The rug goes with a dollar bill because the lady found a dollar while sweeping under the rug. | Non-Unitized |
| 1. **slipper** | The slipper is green because it is for Oscar the Grouch and he's green. | Unitized | The slipper had a dollar bill in it because Cinderella put it there for safekeeping. | Non-Unitized |
| 1. **snow** | The snow is green because it is covered with fallen pine needles. | Unitized | The snow had a dollar bill in it because a dollar fell out of the boy's pocket while he was making a snow angel. | Non-Unitized |
| 1. **stove** | The stove is green because someone was cooking broccoli and spilled it. | Unitized | The stove had a dollar bill on it so the babysitter could buy dinner while the parents were out. | Non-Unitized |
| 1. **tie** | The tie is green because it is part of the uniform for the We Love Lime Jello Club. | Unitized | The tie has a dollar bill pinned to it because the business man was playing pin the tail on the donkey with a picture of his boss and dollar bills. | Non-Unitized |
| 1. **van** | The van is green because it belongs to Shrek. | Unitized | The van had a dollar bill painted on it because it belonged to a store called the Dollar Store, where everything cost a dollar. | Non-Unitized |
| 1. **wave** | The wave is green because it was full of green seaweed. | Unitized | The wave has dollar bills floating on the surface because a shark bit a hole in a boat filled with money. | Non-Unitized |
| 1. **badge** | The badge is red because it belonged to a junior fire fighter. | Unitized | The badge is next to the stop sign because a police man is wearing it and waiting to give tickets to cars that do not stop. | Non-Unitized |
| 1. **bag** | The bag is red because it belongs to Santa Claus. | Unitized | Someone put a bag over the stop sign to trick the drivers. | Non-Unitized |
| 1. **beaver** | The beaver is red because it crawled across a bridge that had wet red paint on it. | Unitized | The beaver goes with a stop sign because it chewed through the sign post to use it to build its home. | Non-Unitized |
| 1. **bucket** | The bucket is red because it goes in the red barn. | Unitized | The bucket was left by the man who painted the stop sign. | Non-Unitized |
| 1. **castle** | The castle is red because it belongs to a king who loves the color red. | Unitized | The castle has a stop sign on the road in front of it so people can stop and take pictures of the beautiful building. | Non-Unitized |
| 1. **elephant** | The elephant is red because it got a bad sunburn. | Unitized | The elephant is standing at the stop sign because it is in a parade and it has to stop so the horses can go by. | Non-Unitized |
| 1. **fountain** | The fountain is red because cherry punch is flowing through it. | Unitized | The fountain is behind the stop sign to keep children from jumping into the fountain to swim with the fish. | Non-Unitized |
| 1. **fox** | The fox is red because it rolled in red mud. | Unitized | The fox ran into the stop sign because it wasn't looking where it was going. | Non-Unitized |
| 1. **jeep** | The jeep is red because it belongs to Elmo. | Unitized | The jeep was in an accident and ran over the stop sign. | Non-Unitized |
| 1. **leaf** | The leaf is red because it is Fall and the leaves are changing colors. | Unitized | The leaf is next to the stop sign because the tree had a broken branch that hangs down in front of the stop sign. | Non-Unitized |
| 1. **net** | The net is red because it helps to attract the crab and lobsters for the fishermen. | Unitized | The net goes with the stop sign because it flew off the back of a fisherman's truck and got tangled around the stop sign. | Non-Unitized |
| 1. **soda** | The soda is red because it is a cherry flavored drink. | Unitized | The soda goes with the stop sign because the boy threw his soda cup out of the car window and it splashed the sign. | Non-Unitized |
| 1. **star** | The star is red because it is on a red fire truck. | Unitized | The star goes with the stop sign because the student put a star sticker on the stop sign. | Non-Unitized |
| 1. **stroller** | The stroller is red because it is painted to look like a wagon. | Unitized | The stroller ran into the stop sign when the babysitter lost her grip on it at the top of the hill. | Non-Unitized |
| 1. **thread** | The thread is red so that it can be used to sew a heart. | Unitized | The thread is wrapped around the stop sign because someone tied a flier about their lost dog to the sign. | Non-Unitized |
| 1. **bell** | The bell goes with a dollar bill because the school asked all the parents to give a dollar so that a new bell could be bought. | Non-Unitized | The bell is green because it is the doorbell for a Greenhouse where they grow plants. | Unitized |
| 1. **bug** | The bug goes with a dollar bill because the little boy decided not to pick up the dollar bill when he saw the large bug on it. | Non-Unitized | The bug is green because it is a grasshopper. | Unitized |
| 1. **canoe** | The canoe goes with a dollar bill because a dollar fell out of the man's pocket while he was rowing around the lake. | Non-Unitized | The canoe is green because it is covered in green leaves that fell out of the tree it was sitting under. | Unitized |
| 1. **fire** | The fire goes with a dollar bill because the campers didn't have any paper to use to make a fire so they used dollar bills instead. | Non-Unitized | The fire is green because it is magical fire made by a wizard. | Unitized |
| 1. **forest** | The forest was covered with dollar bills because Robin Hood was taking money from the rich to give to the poor. | Non-Unitized | The forest is green because there was a heavy rain and all of the plants and leaves are growing. | Unitized |
| 1. **glove** | The glove had a dollar bill stuffed in one of the fingers, which made it hard for the man to put it on. | Non-Unitized | The glove is green because it is used for working in the vegetable garden. | Unitized |
| 1. **key** | The key goes with a dollar bill because it unlocks the pirate’s treasure chest where more money can be found. | Non-Unitized | The key is green because it belongs to the alien's green house. | Unitized |
| 1. **mask** | The mask won a dollar bill as a prize in the costume contest. | Non-Unitized | The mask is green because its wearer is dressed as a piece of celery for Halloween. | Unitized |
| 1. **otter** | The otter is playing with a dollar bill that it found inside of the clam it was eating. | Non-Unitized | The otter is green because it slithered through slime. | Unitized |
| 1. **peach** | The peach is on top of the dollar bill because the mom left a peach and a dollar for her daughter's lunch. | Non-Unitized | The peach is green because it is not ready to eat. | Unitized |
| 1. **piano** | The piano has a dollar bill on top of it because someone paid the piano player to play a special song for the birthday party. | Non-Unitized | The piano is green to match Kermit the Frog who is sitting on it and singing. | Unitized |
| 1. **pool** | The pool has a dollar bill floating in it because a dollar drifted out of the swimmer's shorts while he was playing in the water. | Non-Unitized | The pool is green because it is the home to a family of alligators. | Unitized |
| 1. **rattle** | The rattle goes with a dollar bill because when the baby saw the dad shake a dollar bill, she shook her rattle. | Non-Unitized | The rattle is green because it is for a green baby lizard. | Unitized |
| 1. **sock** | The sock has a dollar bill in it because Santa put it there as a present. | Non-Unitized | The sock is green because the grandmother knitted it for her grandson whose favorite color is green. | Unitized |
| 1. **tire** | The tire on a truck ran over a dollar bill and it got stuck to it. | Non-Unitized | The tire is green because it drove through a park and was covered in grass stains. | Unitized |
| 1. **bean** | The bean goes with a stop sign because when Jack's magic bean was planted it grew into a very large stop sign (not a bean stalk!). | Non-Unitized | The bean is red because it was cooked in tomato sauce. | Unitized |
| 1. **belt** | The belt was hanging on the stop sign because someone found it in the street and wanted to make sure its owner could find it. | Non-Unitized | The belt is red because it is a child's belt with Strawberry Shortcake on it. | Unitized |
| 1. **bowl** | The bowl is next to the stop sign because the boy forgot it there after he fed his dog on their walk. | Non-Unitized | The bowl is red because it was used to hold tomato soup. | Unitized |
| 1. **button** | The button was shaped like a stop sign because it was on a crossing guard's coat. | Non-Unitized | The button is red because it's on Santa's coat. | Unitized |
| 1. **cape** | The cape goes with a stop sign because it belongs to the superhero called Stopman. | Non-Unitized | The cape is red because it belongs to the superhero called Superman. | Unitized |
| 1. **coin** | The coin was next to the stop sign because it rolled down the street and stopped there. | Non-Unitized | The coin is red because it fell in red Jell-O. | Unitized |
| 1. **couch** | The couch goes with the stop sign because it fell off the moving truck and landed next to the sign. | Non-Unitized | The couch is red because someone spilled fruit punch on it. | Unitized |
| 1. **duck** | The duck took a break by the stop sign as it was flying south for the winter. | Non-Unitized | The duck is red because it dressed up like an apple for Halloween. | Unitized |
| 1. **egg** | There is egg on the stop sign because a naughty farmer threw it and it broke on the sign. | Non-Unitized | The egg is red because it came from a red bird. | Unitized |
| 1. **hammer** | The hammer goes with the stop sign because it was used to bang the stop sign into the ground. | Non-Unitized | The hammer is red because it fell in the red paint can. | Unitized |
| 1. **iron** | The iron was put behind the stop sign so kids wouldn't play by it and get burned. | Non-Unitized | The iron turns red when it heats up and is too dangerous to touch because it is hot. | Unitized |
| 1. **milk** | The milk goes with the stop sign because a milk truck crashed into it and the milk spilled all over the stop sign. | Non-Unitized | The milk is red because it has way too much strawberry powder flavoring in it. | Unitized |
| 1. **saddle** | The saddle was next to the stop sign because it was on the back of a horse walking down the street. | Non-Unitized | The saddle is red because the horse is being ridden in a 4th of July parade that is all red, white, and blue. | Unitized |
| 1. **sheep** | The sheep is waiting by the stop sign to cross the street to get to the next field. | Non-Unitized | The sheep is red because the owner dyed it to match the red wagon he gave it rides in. | Unitized |
| 1. **turtle** | The turtle stopped at the stop sign because it liked to watch the cars go by. | Non-Unitized | The turtle is red because kids at the beach painted the shell so they could tell which one it was when it was in a group of other turtles. | Unitized |
